# Hyperfibrinogenemia predicts long-term risk of death after ischemic stroke

**DOI:** 10.1007/s11239-014-1122-1

**Published:** 2014-08-09

**Authors:** Marta Swarowska, Agnieszka Polczak, Joanna Pera, Aleksandra Klimkowicz-Mrowiec, Agnieszka Slowik, Tomasz Dziedzic

**Affiliations:** Department of Neurology, Jagiellonian University Medical College, ul. Botaniczna 3, 31-503 Krakow, Poland

**Keywords:** Stroke, Fibrinogen, Mortality, Outcome

## Abstract

In stroke patients higher levels of plasma fibrinogen are associated with increased risk of unfavourable functional outcome and short-term mortality. The aim of our study was to determine the relationship between plasma fibrinogen level and long-term risk of death in ischemic stroke patients. Seven hundred thirty six patients (median age 71; 47.1 % men) admitted to the stroke unit within 24 h after stroke were included. Plasma fibrinogen level was measured on day 1 of hospitalisation. Hyperfibrinogenemia was defined as plasma fibrinogen concentration >3.5 g/L. The maximal follow-up period was 84 months. Hyperfibrinogenemia was found in 25.0 % of patients. On multivariate logistic regression analysis, after adjustment for age, stroke severity, atrial fibrillation, smoking, white blood cell count, fever, in-hospital pneumonia and hyperglycemia, hyperfibrinogenemia was associated with increased case fatality (HR 1.71, 95 % CI 1.29–2.26, *P* < 0.01). Hyperfibrinogenemia predicts the long-term risk of death in ischemic stroke patients.

## Introduction

Fibrinogen is a coagulation factor, a major determinant of plasma viscosity and key factor for platelet activation. It belongs also to acute phase proteins. A numerous studies have demonstrated that plasma fibrinogen level is consistently and independently from traditional risk factors related to cardiovascular risk [1]. Fibrinogen has emerged as an important additional marker for stratification of cardiovascular event risk. The analysis compromising participant data from 52 prospective studies showed that the assessment of fibrinogen in people at intermediate risk for cardiovascular event could help prevent one additional event over a period of 10 years for every 400–500 people so screened [2].

In stroke patients higher levels of plasma fibrinogen are associated with increased risk of unfavourable functional outcome [3–8], short-term mortality [3–5, 9] and new cardiovascular events [10–12].

Much less is known about the association between hyperfibrinogenemia and long-term mortality after stroke. In one study, the ischemic stroke patients with hyperfibrinogenemia were more likely to die after 12 months than those with normal plasma fibriniogen and the increased fibrinogen concentration was an independent predictor of death [3]. From clinical point of view it is important to determine if increased plasma fibrinogen level is related to long-term mortality after stroke. First, fibrinogen could serve as a biomarker to identify patients who are at risk of death. Such patients may require careful monitoring and more aggressive secondary prevention. Second, fibrinogen can be considered as potential therapeutic target, because its level could be reduced by smoking cessation, diet, physical activity and some drugs.

The aim of our study was to determine the relationship between plasma fibrinogen level and long-term risk of death in ischemic stroke patients.

## Materials and methods

We retrospectively analysed the prospectively collected data on prognosis in stroke patients. The consecutive patients with first-ever ischemic stroke admitted to our stroke unit within 24 h after stroke onset were eligible for the study. The patients were recruited to the study between November 2004 and October 2007. The only exclusion criterion was the lack of patient’s consent for participation in the study.

All patients underwent head CT scan within 24 h after stroke onset. Stroke severity on admission was assessed using National Institute of Health Stroke Scale (NIHSS).

Arterial hypertension was diagnosed when its presence was documented in medical records or when at least two readings of blood pressure were ≥140 mmHg (systolic) or ≥90 mmHg (diastolic) after the acute phase of stroke. The diagnosis of diabetes mellitus was made when (1) the patient had the recognized diabetes mellitus before stroke as written in medical records and/or took hypoglycemic drugs before stroke; (2) fasting plasma glucose measured on day 6–10 was ≥7.0 mmol/L or fasting plasma glucose was 6.1–6.9 mmol/L and 2 h plasma glucose was ≥11.1 mmol/L after oral glucose tolerance test. A patient was defined as a smoker if there was a history of cigarette smoking during the last 5 years.

Plasma fibrinogen level was determined on day 1 using modified Clauss method (Dade Behring, Marburg, Germany). Hyperfibrinogenemia was defined as plasma concentration >3.5 g/L.

The follow-up was up to 84 months with the minimal duration of 12 months. Information about death was taken from the town registry.

The study protocol was approved by the local Bioethics Committee and each participant gave the informed consent.

The *χ* ^2^ test was used to compare proportions and Mann–Whitney’s test to compare continuous variables between groups. Values of less than 0.05 were considerate to indicate statistical significance. Log-rank test was used to compare survival between the patients with hyperfibrinogenemia and those with normofibrinogenemia. Cox’s proportional hazard models were used to find the independent predictors of death on univariate and multivariate analysis. The variables with *P* < 0.10 on univariate analysis were included in multivariate analysis. The calculations were performed using the program STATISTICA for Windows (version 10, Statsoft, Poland).

## Results

Seven hundred thirty six patients fulfilled the inclusion criteria (median age 71, interquartiles 62–79; 47.1 % men). Hyperfibrinogenemia was found in 184 (25.0 %) patients. Table 1 shows the characteristic of patients with hyperfibrinogenemia and those without it.

**Table 1:** The baseline characteristics of the patients with and those without hyperfibrinogenemia

	Patients with normofibrinogenemia (N = 552)	Patients with hyperfibrinogenemia (N = 184)	*P*
Age, median (IQ)	70.0 (63.0–78.0)	71.5 (62.0–81.0)	0.32
Men, n (%)	243 (44.0)	104 (56.5)	<0.01
Hypertension, n (%)	375 (67.9)	124 (67.4)	0.89
Diabetes mellitus, n (%)	115 (20.8)	37 (20.1)	0.83
Previous myocardial infarction, n (%)	64 (11.6)	33 (17.9)	0.03
Atrial fibrillation, n (%)	127 (23.0)	30 (16.3)	0.05
Smoking, n (%)	145 (26.3)	55 (29.9)	0.34
NIHSS score on admission, median (IQ)	11.0 (6.8–17.0)	12.0 (8.0–17.0)	0.18
Systolic blood pressure on admission (mmHg), median (IQ)	160 (140–180)	160 (140–180)	0.83
Diastolic blood pressure on admission (mmHg), median (IQ)	90 (80–100)	90 (80–100)	0.26
Glucose on admission >7.0 mmol/L, n (%)	224 (40.5)	78 (42.4)	0.65
Fasting glucose >6.1 mmol/L, n (%)	225 (40.8)	82 (44.6)	0.42
Total cholesterol (mmol/L), median (IQ)	5.3 (4.5–6.2)	5.3 (4.5–6.4)	0.93
Body temperature >37.5 °C during first 48 h, n (%)	122 (22.1)	57 (30.9)	0.02
WBC count >10 000/μL, n (%)	150 (27.2)	77 (41.8)	<0.01
In-hospital pneumonia, n (%)	58 (10.5)	17 (9.2)	0.62

The patients with hyperfibrinogenemia more often were male, suffered from previous myocardial infarction, had white blood cell count >10,000/μL and the body temperature >37.5 °C during first 48 h of stroke.

**Fig. 1:**
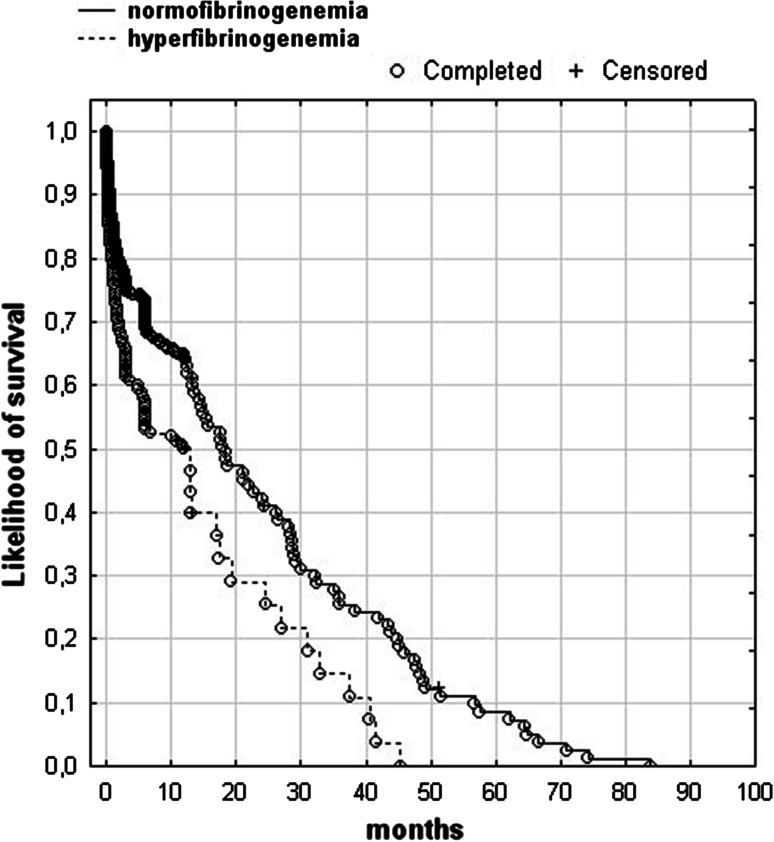
Kaplan–Maier survival curves for the patients with and those without hyperfibrinogenemia

Figure 1 shows Kaplan–Meier’s curves of survival for the patients with hyperfibrinogenemia and the patients with normofibrinogenemia. The patients with hyperfibrinogenemia had shorter time of survival than those with normofibrinogenemia (*P* < 0.01, log-rank test).

Table 2 shows independent predictors of death on univariate analysis.

**Table 2:** The predictors of death on univariate analysis

	HR, 95 %CI	*P* value
Age (increase per unit)	1.03 (1.02–1.04)	<0.01
NIHSS score on admission (increase per unit)	1.09 (1.07–1.11)	<0.01
Atrial fibrillation	1.75 (1.37–2.25)	<0.01
Smoking	0.69 (0.54–0.89)	<0.01
WBC count >10 000/μL	1.46 (1.17–1.83)	<0.01
Body temperature >37.5 °C during first 48 h	2.03 (1.60–2.58)	<0.01
In-hospital pneumonia	2.53 (1.90–3.37)	<0.01
Hyperfibrinogenemia	1.62 (1.28–2.07)	<0.01
Fasting glucose >6.1 mmol/L	1.73 (1.38–2.18)	<0.01

Hyperfibrinogenemia was associated with risk of death both on univariate (HR 1.62, 95 % CI 1.28–2.07, *P* < 0.01) and multivariate analysis (HR 1.71, 95 % CI 1.29–2.26, *P* < 0.01). Other independent predictors of death on multivariate analysis were: age (HR 1.03, 95 % CI 1.01–1.04, *P* < 0.01), NIHSS score (HR 1.08, 95 % CI 1.05–1.10, *P* < 0.01), atrial fibrillation (HR 1.62, 95 % CI 1.20–2.18, *P* < 0.01), white blood cell count count >10,000/μL (HR 1.32, 95 % CI 1.02–1.71, *P* = 0.03) and the body temperature >37.5 °C during first 48 h of stroke (HR 1.48, 95 % CI 1.13–1.92, *P* < 0.01).

## Discussion

In our study hyperfibrinogenemia was associated with long-term risk of death after ischemic stroke and this relationship was independent from age, stroke severity, and selected inflammation-related variables (fever, leukocytosis, in-hospital pneumonia). New information provided by our study is that in relatively large cohort of unselected ischemic stroke patients hyperfibrynogenemia predicts mortality beyond 1 year.

The results of a few studies indicate that hyperfibrinogenemia observed in acute stroke persists even 1 year after the event. It suggests that the stimuli for fibrinogen synthesis are still active even months after cerebral infarction [13, 14]. Levels of fibrinogen are influenced by genotype and therefore persistent hyperfibrinogenemia could be related to variation in genomic sequences [15, 16]. Finally, the elevated levels of fibrinogen observed months after stroke might reflect pre-stroke hyperfibrinogenemia which is considered as a risk factor for stroke [17, 18].

Our findings should be interpreted in light of the results of the studies investigating the relationship between fibrinogen and mortality in non-stroke populations. In large individual participant meta-analysis, moderately strong association was found between plasma fibrinogen and the risk of vascular and non-vascular mortality in healthy middle age adults [18]. In EPIC-Norfolk study including 16,850 participants aged 40–79, fibrinogen level predicts long-term risk of death after adjustment of cardiovascular risk factors [19]. Higher fibrinogen concentration was also associated with increased risk of death in patients with unstable angina [20], diabetes mellitus [21] or renal failure [22, 23]. Taking together, these results suggest that plasma fibrinogen level is a non-specific marker of mortality and bring the causal link between hyperfibrinogenemia and increased risk of death in question.

It remains unclear if hyperfibrinogenemia in stroke patients is only a marker of mortality or a real culprit. The assessment of any causal relationship of fibrinogen level with mortality in stroke patients will require randomized trials of selective fibrinogen-lowering drugs or Mendelian randomization studies assessing genetic determinants of fibrinogen level.

Fibrinogen levels can be reduced by lifestyle modification (for example, smoking cessation, regular exercises, diet, etc.) or medication (for example, fibrates). Taking into account the persistence of hyperfibrinogenemia after stroke and its relationship to death, any interventions aimed to decrease post-stroke mortality by lowering fibrinogen levels should be long-lasting.

In conclusion, hyperfibrinogenemia predicts the long-term risk of death in ischemic stroke patients.
